# A Non-Destructive Methodology for the Viscoelastic Characterization of Polymers: Toward the Identification of the Time–Temperature Superposition Shift Law

**DOI:** 10.3390/s23229213

**Published:** 2023-11-16

**Authors:** Aleksandr Sakhnevych, Raffaele Maglione, Francesco Timpone

**Affiliations:** Department of Industrial Engineering, University of Naples Federico II, 80125 Naples, Italy; raffaele.maglione@unina.it (R.M.); francesco.timpone@unina.it (F.T.)

**Keywords:** viscoelasticity, material testing, non-destructive methodology, time–temperature superposition, WLF equation

## Abstract

Polymers find widespread applications in various industries, such as civil engineering, aerospace, and industrial machinery, contributing to vibration control, dampening, and insulation. To accurately design products that are able to predict their dynamic behavior in the virtual environment, it is essential to understand and reproduce their viscoelastic properties via material physical modeling. While Dynamic Mechanical Analysis (DMA) has traditionally been used, innovative non-destructive techniques are emerging for characterizing components and monitoring their performance without deconstructing them. In this context, the Time–Temperature Superposition Principle (TTSP) represents a powerful empirical procedure to extend a polymer’s viscoelastic behavior across a wider frequency range. This study focuses on replicating an indentation test on viscoelastic materials using the non-destructive Viscoelasticity Evaluation System evolved (VESevo) tool. The primary objective is to derive a unique temperature–frequency relationship, referred to as a “shift law”, using characteristic curves from this non-invasive approach. Encouragingly, modifying the device setup enabled us to replicate, virtually, three tests under identical initial conditions but with varying indentation frequencies. This highlights the tool’s ability to conduct material testing across a range of frequencies. These findings set the stage for our upcoming experiment campaign, aiming to create an innovative shift algorithm from at least three distinct master curves at specific frequencies, offering a significant breakthrough in non-destructive polymer characterization with broad industrial potential.

## 1. Introduction

Viscoelastic materials are a class of substances that exhibit both viscous (flow-like) and elastic (reversible deformation) properties when subjected to mechanical stresses. These materials play a pivotal role in various applications, ranging from aerospace engineering to biomedical devices. One area where their properties have an exceptionally profound impact is the automotive industry, specifically in the design and performance of tires [1,2]. Tires play a significant role in vehicular dynamics by serving as the essential interface between a vehicle and the road surface. Their performance has broad implications, affecting not only a vehicle’s handling, safety, and fuel efficiency but also ride comfort and environmental considerations. Furthermore, the investigation of the tire tread viscoelastic properties is of fundamental importance for the search for new solutions in tire manufacturing and the development of proper contact mechanics models necessary to improve the prediction of the interactions that pneumatic tires exchange with the road surface [3].

Therefore, knowing the properties of these materials is of considerable importance although it is not as simple as it might seem, given their unique response that involves a blend of purely elastic and viscous processes [4,5]. Moreover, this response is heavily dependent on both time and temperature [6,7]. Understanding how these properties evolve in relation to temperature is a critical aspect in numerous applications, including the tire performance prediction domain, where forecasting the thermal characteristics of tires continues to be an ongoing and challenging pursuit for researchers [8,9].

Dynamic mechanical analysis [10] is a typical characterization technique employed for the evaluation of the viscoelastic response of the tire tread surface, although the destruction of the compound and long testing times are necessarily required. In this scenario, the development of non-invasive testing methods represents a complex but engaging challenge that involves the most disparate sectors, from industries to academic research [11,12,13]. For this reason, a new innovative methodology, known as VESevo, was developed by the applied mechanics research group of the Department of Industrial Engineering at the University of Naples Federico II. This innovative technique performs a non-destructive characterization through a rod indentation on the material, providing the viscoelastic curves, in terms of storage modulus and loss factor, at a reference frequency of 1 Hz. VESevo can track variations due to thermal cycles and aging and monitor the time evolution of the viscoelastic properties of polymers without causing damage [14].

Depending on the field of pneumatic tire application and the range of angular velocities generated by the wheel, it becomes useful to extend the knowledge of the compound mechanical behavior by analyzing the evolution of its temperature-dependent response in a higher-frequency range compared to the reference one [15,16]. The construction of an equivalent master curve at a new reference frequency requires the application of the Time–Temperature Superposition Principle in order to obtain the horizontal shift factors that allow the reconstruction of the master curve over a wide frequency range.

There are several shift laws describing the variation in the shift factors with temperature, such as the Arrhenius law or other methodologies based on the use of the first derivative of elastic modulus curves [17], but among all, the most widespread and most suitable in a vast range of temperatures is the Williams–Landel–Ferry (WLF) equation [18,19,20,21].

However, a persistent challenge that remains is the assessment of the WLF coefficients using non-invasive techniques. Currently, these coefficients are typically determined through destructive experiments at various frequencies using the DMA methodology [22,23], which poses a significant limitation that needs to be addressed. To this end, in the current work, a new empirical procedure, consisting of changing the frequency of the indentation phenomenon by varying the mass of the VESevo indenter, is conceptualized. A simulation model is developed to virtually change the design parameters of the VESevo device in order to perform a sensitivity analysis of the main model parameters. The research idea is to reconstruct at least three master curves at three different frequencies that show the evolution of the viscoelastic properties with temperature. Using these, the shift factors are evaluated, and a shift law that allows the complete characterization of the material in a completely non-invasive way is extrapolated.

This simulation study, validated by appropriate experimental tests, illustrates the feasibility of obtaining these curves by changing the values of the indenter masses, demonstrating that VESevo could serve as a valid tool for identifying a novel shift algorithm without any component alteration.

## 2. Mechanical Behavior of Viscoelastic Materials

Polymers can be classified as viscoelastic materials due to their particular behavior, which is halfway between an elastic solid and a viscous fluid. Typically, the viscoelastic properties of these materials are investigated with dynamic experiments imposing a sinusoidal oscillating input force (or deformation), causing a sinusoidal stress (or strain) to be applied to the sample, which generates a sinusoidal strain (or stress) that can be measured [24].

**Figure 1:**
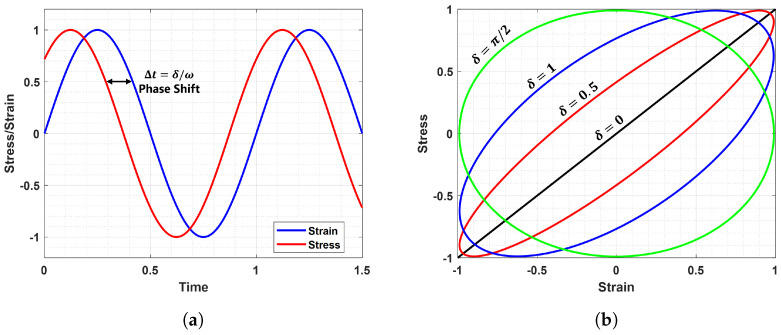
(**a**) Phase lag between stress and strain (**b**) Stress-Strain curves at different phase angles. 0<δ<π/2.

For this class of materials, a signal shift between input and output occurs with a phase angle between 0 and 90, as shown in Figure 1. In particular,Figure 1a shows how the strain response is an oscillation at the same frequency as the stress but lags by a phase angle δ. Alternatively, this phase shift can be seen with the Lissajous curves in Figure 1b: when δ=0, the stress–strain curve is a straight line and no dissipative phenomenon occurs, reflecting the perfectly elastic material response; conversely, when δ≠0, a lag between cause and effect exists and an elliptical hysteresis loop appears.

The input solicitation and the output response are related to each other via a transfer function that physically contains all the information about the viscoelastic properties of the sample under analysis. This peculiar function is called a complex dynamic modulus and is mathematically defined as:

(1) $$E^{*}\left(i\omega \right)=E^{\prime }\left(\omega \right)+iE^{\prime \prime }\left(\omega \right)$$

In other words, this dynamic viscoelastic function is characterized by real and imaginary parts, each of which has a specific physical meaning [25]. The first one is associated with the material’s capacity to respond to external loads without any energy dissipation, resulting in a full elastic recovery. For this reason, $E^{\prime }\left(\omega \right)$ is related to energy storage mechanisms, and it is known as the storage modulus. Moreover, $E^{\prime \prime }\left(\omega \right)$ describes completely irreversible processes that are not recoverable when external loads are removed; therefore, it is called the loss modulus [26,27,28,29,30]. The ratio between these two dynamic functions is equal to:

(2) $$tan\delta \left(\omega \right)=\frac{E^{\prime \prime }\left(\omega \right)}{E^{\prime }\left(\omega \right)}$$

It is defined as the loss factor, that is, a dimensionless measure of the viscoelastic damping of polymeric materials, which describes their capability to dissipate energy, as highlighted in a typical stress–strain curve (Figure 1b). The area enclosed by the loop physically represents the amount of energy dissipated as heat in a loading–unloading cycle, commonly referred to as a hysteresis cycle, which is a direct consequence of the lag that exists between cause and effect for these materials [7].

These viscoelastic properties of polymers, such as the tire tread compound, are typically experimentally evaluated via dynamic mechanical analysis. This characterization technique allows for obtaining much information about the polymeric sample, subjecting it to bending or torsional forces at different temperatures within a frequency range from 0.1 Hz to 100 Hz [10]. Different clamps can be employed in a DMA, depending on the material tested and the dynamic viscoelastic function investigated. The most common are three-point bending and dual cantilever for dynamic modulus $E^{*}$ evaluation and parallel plates in torsional mode for complex shear modulus $G^{*}$.

**Figure 2:**
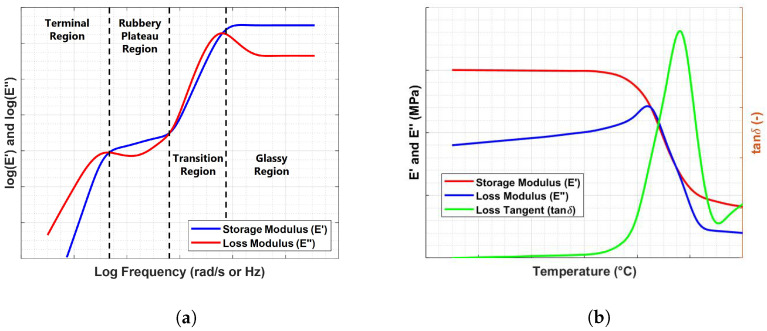
(**a**) Viscoelastic moduli as a function of frequency (**b**). Viscoelastic moduli as a function of temperature.

Generally, two kinds of test modes can be carried out to characterize the viscoelastic behavior of polymers: temperature sweep and frequency sweep tests. The former provides temperature-dependent viscoelastic properties since the frequency of the oscillating input is kept constant; the latter is useful to investigate frequency effects on viscoelastic materials at a specific temperature. A typical output of these two test modes is illustrated in Figure 2 in terms of dynamic viscoelastic functions.

Polymeric materials exhibit a constant frequency response in the borderline cases of very low (rubbery plateau region) and high-frequency (glassy region) values, whereas a strong frequency dependence occurs in the linear viscoelastic region where peaks in loss factor and loss modulus curves emerge [6]. The same behavioral regions can be investigated in temperature sweep mode. The equivalence relation between time (or frequency) and temperature is formalized via the Time–Temperature Correspondence Principle, which allows the development of an empirical procedure, known as the Time–Temperature Superposition Principle, for the construction of master curves. Different relations that link time (or frequency) and temperature have been developed, including the well-known Williams–Landel–Ferry equation, which was obtained starting from the theoretical concept of free volume [27]. This relation can be mathematically expressed via the following empirical equation:

(3) $$loga_{T}=\frac{-C_{1}\left(T-T_{0}\right)}{C_{2}+T-T_{0}}$$

where $C_{1}$ and $C_{2}$ are the fitting coefficients of the WLF relationship, $T_{0}$ is the reference temperature of the master curve, and $a_{T}$ is the shift factor that satisfies the following equality relationship:

(4) $$E^{*}\left(\omega ,T\right)=E^{*}\left(\xi ,T_{0}\right)$$

where $\xi =\omega_{T}a_{T}\left(T,T_{0}\right)$ is the reduced frequency. When the glass transition is selected as the reference temperature, the constants C1 and C2 are assigned values of 17.4 and 51.6, respectively. However, in real-world applications, the reference temperature often differs from the glass transition temperature. Consequently, destructive methods become essential for determining the values of the WLF constants. This limitation forms the basis for the research question addressed in this study.

## 3. Non-Destructive Viscoelasticity Evaluation: VESevo

To overcome the limitations of the conventional destructive characterization techniques for viscoelastic materials, the Vehicle Dynamics Research Group of the Department of Industrial Engineering at the University of Naples Federico II developed the innovative device VESevo. This device represents a valid solution capable of testing tire tread compounds and almost any material that exhibits viscoelastic behavior in a completely non-destructive way. It allows one to characterize the material’s viscoelastic properties and their variations due to cooling/heating thermal cycles as well as monitor their time evolution as a result of wear and aging phenomena without altering the tested component [14].

**Figure 3:**
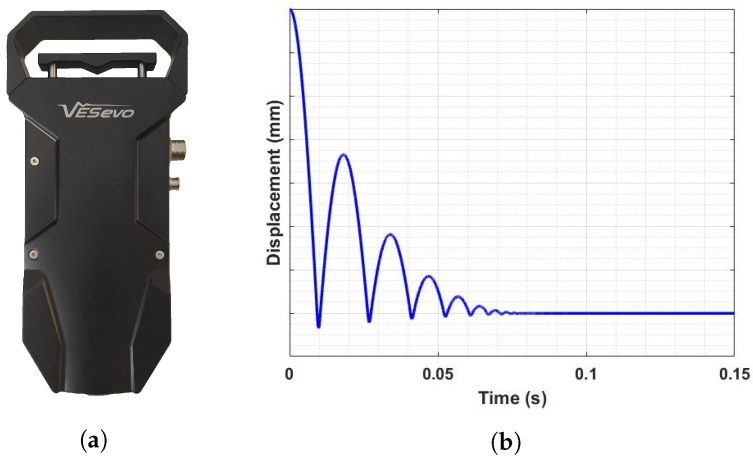
(**a**) VESevo device (**b**). Raw signal of the rod displacement.

The VESevo device houses a rod–spring mechanism that consists of a steel rod with a semi-spherical indenter and a spring element. A suitable guide allows the steel rod to slide and bounce freely once it hits the sample surface. An innovative patented unhooking system is implemented to guarantee that the motion of the rod always starts from the same initial position. The rod displacement is measured by means of an optical laser sensor whereas a compact-size IR pyrometer monitors the tread surface temperature (Figure 3a).

The acquisition phase consists of lifting the trigger of the device, which allows the rod to be raised to the release point. The unhooking system then drops the rod, which slides along the guide and impacts and bounces on the surface of the compound sample, while the optical sensor measures the rod displacement. In Figure 3b, a typical raw signal of the displacement curve during a single acquisition is shown for a specific measured temperature.

The displacement curve exhibits three different phases that are essential for the evaluation of specific physical magnitudes related to the viscoelastic behavior: the initial monotonically decreasing curve branch reflects the free fall of the steel rod followed by the sinking of the indenter, corresponding to the minimum point of the acquisition curve. In the second phase, the rod reaches the maximum point of the curve after the first indentation as a result of its interaction with the tread surface. The last phase is characterized by a stabilized rod displacement value corresponding to indenter–sample contact when the rebound phenomenon is completely extinct.

The experimental setup is straightforward, requiring the VESevo device, access to an electrical network, and a computer for connecting the device. After positioning the sample or object to be tested, an operator manually lifts the trigger, releasing the rod automatically. Following each test, the rebound curve is displayed on the PC’s graphical interface. After completing all tests, the data are processed to obtain the desired properties. Additional tools for heating or cooling the sample, as well as adapters for addressing specific shape-related challenges in individual samples, can be employed as needed.

In order to investigate the temperature dependence of the compound viscoelastic response, the displacement curves are acquired over the entire operating temperature range. Typically, the VESevo test session is carried out using the following standard testing procedure [14]:

- Several acquisitions at ambient temperature;
- Cooling to −30 °C employing a climatic cell or a freezing spray and then performing VESevo acquisitions during the natural heating process up to ambient temperature;
- Forced heating up to 100 °C through a thermal blanket or a professional heating gun and then carrying out acquisitions down to ambient temperature.

**Figure 4:**
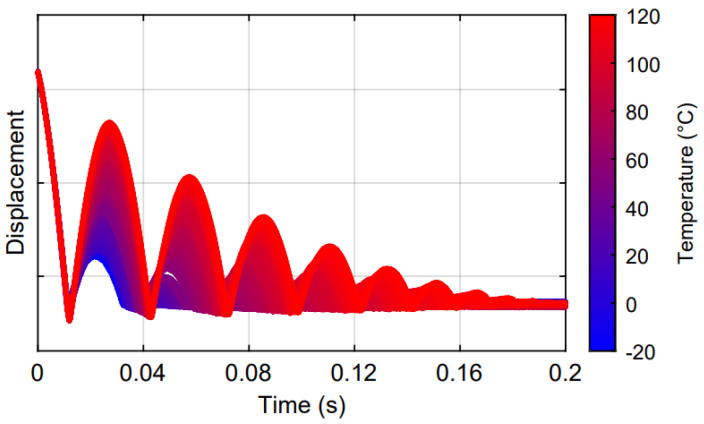
Displacement curves under different temperatures of the tire tread surface.

Figure 4 clearly highlights how the temperature strongly affects the rod–sample interaction and, consequently, the rebound phenomenon both in terms of the amplitude and bounce number, reflecting the natural viscoelastic response of the tread compound as a function of temperature: at low temperatures, high energy dissipation occurs and the material behaves as a glassy solid. Conversely, when the compound is heated up to about 100 °C, a progressive decrease in energy dissipation is observed, with a resulting increase in rebound number and a rubbery behavior clearly emerges.

The rod displacement raw signals, acquired throughout the temperature range, contain all the information necessary to extrapolate, through a sophisticated post-processing algorithm, a series of physical temperature-dependent parameters with which it is possible to define specific mathematical relationships for the evaluation of the viscoelastic properties and the construction of the full master curves. Particularly, the storage and loss moduli can be defined using the following functional relations:

(5) $$\begin{array}{c} E_{1}=f\left(A_{c},T,K_{c}\right)\\ E_{2}=f\left(A_{c},\omega ,T,S_{c}\right) \end{array}$$

where $A_{c}$ is the effective contact area between the semi-spherical indenter and the compound, ω is the solicitation frequency linked to the single VESevo test, *T* is the compound temperature, and $K_{c}$ and $S_{c}$ are the equivalent contact stiffness and damping coefficient.

**Figure 5:**
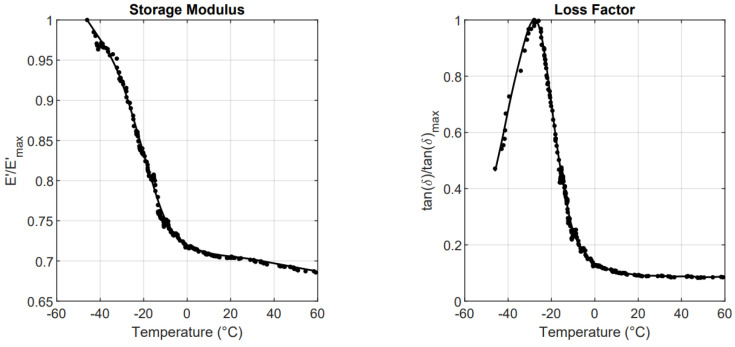
Viscoelastic master curves of a tire tread compound at 1 Hz.

The loss factor is calculated as the ratio of $E_{2}$ and $E_{1}$. The resulting master curves are shown in Figure 5.

## 4. Methodology: Virtual Model, Sensitivity Analysis, and Experimental Validation

The TTSP represents a powerful empirical procedure to extend the knowledge of the viscoelastic behavior of the polymers in a frequency range wider than the experimentally investigable one. However, the actual configuration of the VESevo device, based on a single indenter mass, does not allow us to uniquely identify the temperature–frequency relation of the material under analysis. In fact, it provides the master curve at only one reference frequency of 1 Hz. Therefore, a preliminary simulation study was conducted to perform a sensitivity analysis in order to investigate the extent of the effects that variations in the main design parameters of the VESevo device have on the rod dynamics. In particular, the aim was to discern whether it is possible to acquire noticeable distinct dynamic indenter responses, thereby enabling the generation of a minimum of three viscoelastic master curves, which is fundamental for the development of an innovative shifting algorithm.

The simulation model was developed, starting from the components CAD (Computer Aided Design), in the Simulink environment and allows for virtually replicating the real VESevo device and changing the physical parameters of different device components without making any modifications to the actual device. Thus, with a simple personal computer and a relatively low computational cost, it is possible to build several VESevo configurations.

**Figure 6:**
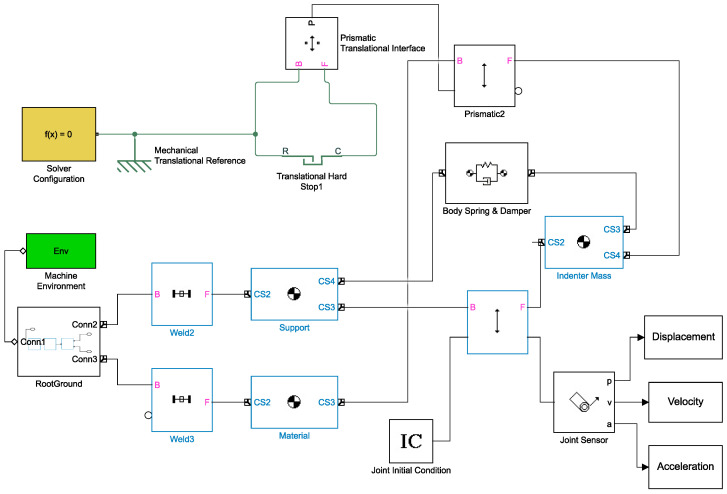
Virtual model of the VESevo device indenting a viscoelastic material.

The virtual model is schematically shown in Figure 6. The machine environment block (green in the block diagram) defines the mechanical simulation environment for the system attached to this block: it allows for setting a series of global options, including gravity, system dimensionality, and analysis mode. All blocks that are identified by this symbol 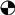 (center of gravity) represent the body blocks, which are rigid bodies with a specific mass, coordinates of their center of gravity, and other user-defined properties. These bodies can be rigidly connected, or they can move with respect to each other with translational motion; in the first case, a weld block was used (it represents 0 degrees of freedom (DOF)), while in the second case, a prismatic block was employed (it represents 1 translational DOF). Each body block was identified using a label showing the name of the real VESevo component that the body block virtually replicates.

Two other important block elements are the “Body Spring & Damper” and the “Translational Hard Stop” blocks. The first one simulates the rod–spring system of the VESevo device. It includes three important parameters to be set: the spring constant, the spring natural length, and a damping coefficient; the latter takes into account the friction phenomena between the rod and the relative guide.

The Translational Hard Stop block recreates the physical phenomenon by which the rod bounces on the sample surface when it comes into contact with the surface itself; this interaction is simulated using a slider (the rod) and a hard stop (the sample under analysis). The hard stop is represented as a spring–damper system, taking into account the elastic interaction between the slider and the stop and the dissipative phenomena. Therefore, it is necessary to set a contact stiffness and a damping coefficient; these two parameters reproduce the elastic response and the dissipative properties of the material under analysis (represented with a simple linear viscoelastic model). Finally, two joint blocks have been implemented: a joint initial condition that simulates the initial drop height of the rod and a joint sensor that can measure the position and velocity of the rod and provide the typical output signals, displacement, and velocity signals.

The constructed model is parametric, meaning it allows for adjustments in various parameters to replicate the design characteristics of the real system without the need for any physical alterations. Additionally, it offers the capability to replicate various material responses that change the hard stop characteristics.

In this research, the approach used to study how the different features influence the system dynamics involves systematically adjusting individual model parameters and observing the corresponding system responses. However, the focus was mainly on investigating the effects of the indenter mass on the rod’s rebound curve and, consequently, the viscoelastic properties determined through the VESevo device’s algorithm.

Ultimately, to guarantee the fidelity of the virtual model and, by extension, the ability to draw valid real-world conclusions, an experimental testing campaign was conducted. This campaign involved an analysis of the outcomes generated via the newly designed components and their subsequent validation against the simulated data.

## 5. Results

As mentioned above, the analysis was conducted by varying the indenter mass while the rod drop height, spring constant, and material characteristics were held constant. The initial challenge encountered in this research endeavor was the discerning selection of mass values, a task that entailed a thorough evaluation of the results obtained when varying mass values, with a particular emphasis on indentation frequency, alongside a comprehensive analysis of overall dimensions and cost factors. Subsequently, after careful consideration, three specific mass values were meticulously chosen for the study, denoted as $m_{1}$, $m_{2}$, and $m_{3}$. Their value can not be stated explicitly, but it is sufficient to say that they respect the following conditions:

(6) $$\begin{array}{c} m_{2}\approx 3.22m_{1}\\ m_{3}\approx 6.74m_{1} \end{array}$$

**Figure 7:**
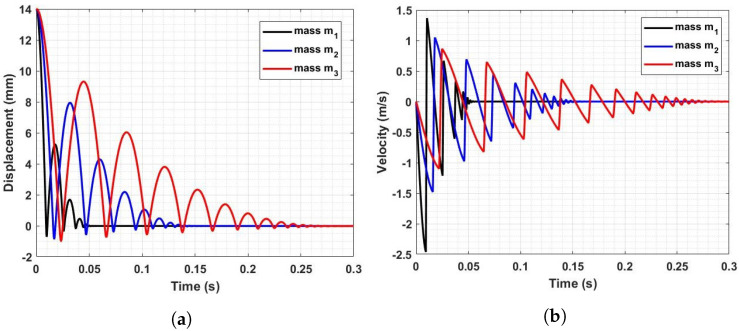
(**a**) Rod displacement signal as a function of time for the three indenter masses. (**b**) Rod velocity signal as a function of time for the three indenter masses.

The displacement raw signals obtained from the simulation, as illustrated in Figure 7a, clearly show that changes in indenter mass produce strong effects on the rod dynamics, both in terms of the number of rebounds and their amplitude. Notably, these curve features increase with larger indenter mass; consequently, it is necessary to set a longer acquisition time to ensure that the rebound phenomenon is completely extinguished within such a time limit.

**Figure 8:**
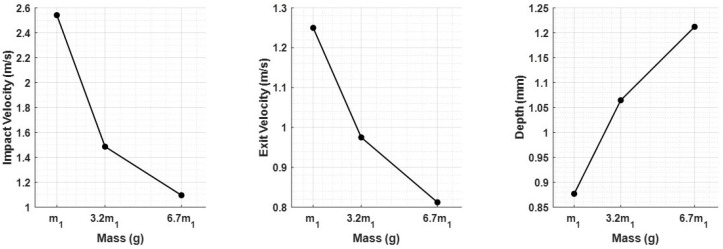
Feature trends as a function of the indenter mass.

The raw signals were then processed with the VESevo data processing algorithm in order to obtain the typical physical parameters. As shown in Figure 7b, the indenter mass variation also has an effect on pre- and post-indentation velocities. In detail, Figure 8 shows how both the impact speed and the exit speed (material–rod detachment) decrease as mass increases, and this has an effect on the indentation depth, which increases with the mass.

**Figure 9:**
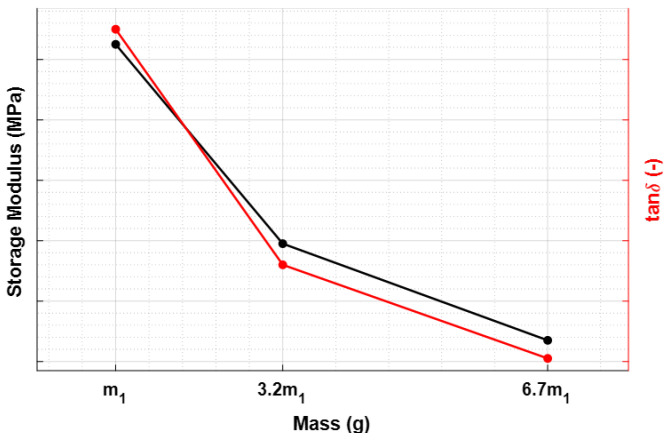
Dynamic viscoelastic properties as a function of the indenter mass.

Since these physical parameters play an important role in the mathematical formulation of dynamic viscoelastic functions, their variations affect the storage modulus and loss factor trends, as depicted in Figure 9.

Consequently, a challenge arises in achieving the uniform viscoelastic characterization of materials, as it depends on the three masses that strongly influence the impact velocity.

**Figure 10:**
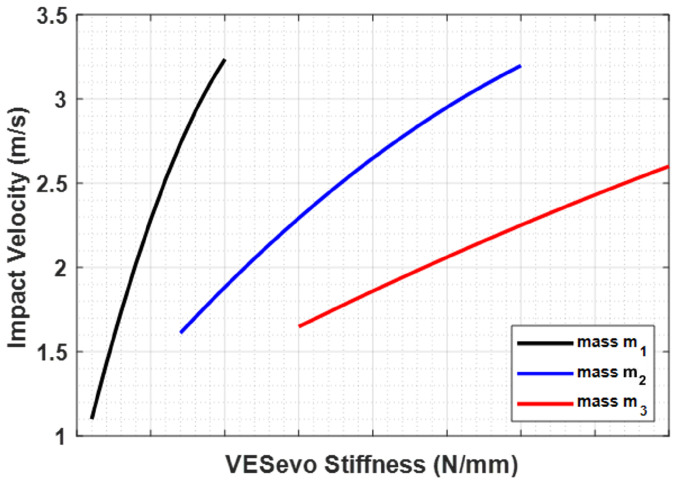
Impact velocity–$K_{VES}$ curves for each indenter mass.

This issue stems from the fact that, in these experiments, the elastic component of the rod–spring system in the virtual VESevo remained consistently constant. In order to solve this problem, the VESevo spring was redesigned to determine the optimal spring constant value, ensuring a constant impact velocity regardless of the indenter mass used. To this end, the simulation model was used to obtain several spring constant values for each indenter mass and to build impact velocity vs. VESevo stiffness ($K_{VES}$) curves, as depicted in Figure 10.

**Figure 11:**
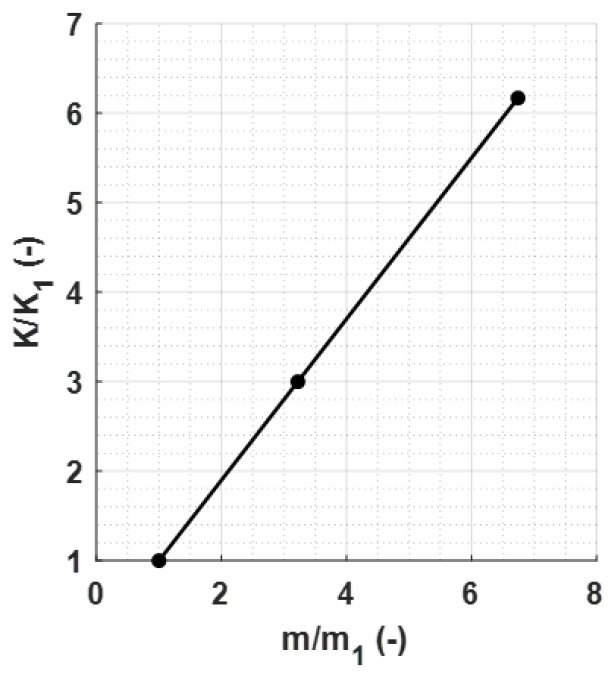
Normalized spring constants as a function of the normalized indenter masses for a target velocity of 2.3 m/s.

From these curves, the $K_{VES}$ values needed to achieve a target velocity of 2.3 m/s were extrapolated. The results are reported in Figure 11.

The normalized values were calculated by dividing each indenter mass by $m_{1}$ and each value of the corresponding spring constant by $K_{1}$.

**Figure 12:**
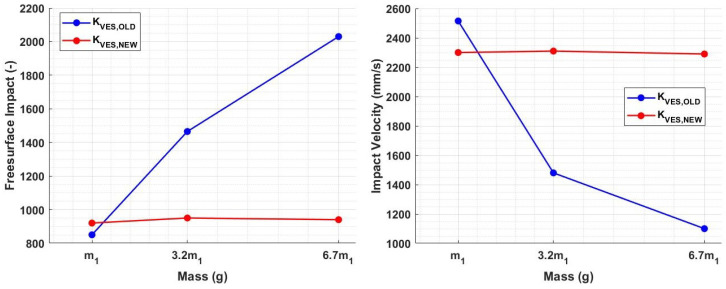
New values of impact-free surface and velocity as a function of the material stiffness for each indenter mass.

By employing these new spring constant values and performing the analysis again, the impact-free surface and velocity remained constant and independent of the indenter mass, as depicted in Figure 12.

With these changes, the energy contribution of the indenter impacts the tread surface; thus, the impact velocity is kept constant, regardless of the employed indenter mass, ensuring complete and correct viscoelastic characterization.

**Figure 13:**
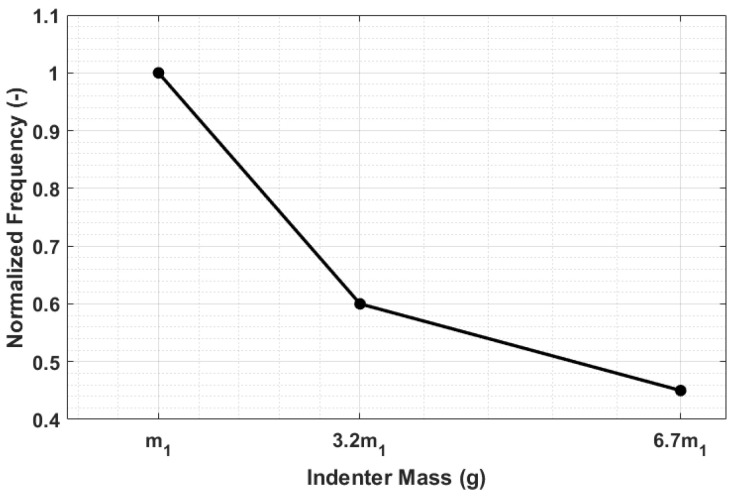
Normalized frequency of the indentation phenomenon as a function of the semi-spherical indenter mass.

Finally, in order to evaluate if the different rod dynamics responses have a noticeable effect on the frequency of the indentation phenomena, the normalized frequencies corresponding to the tests carried out with the three different masses were compared. The results shown in Figure 13 highlight that by employing the three different masses of the semi-spherical indenter, three distinct values of the indentation frequency are obtained.

**Figure 14:**
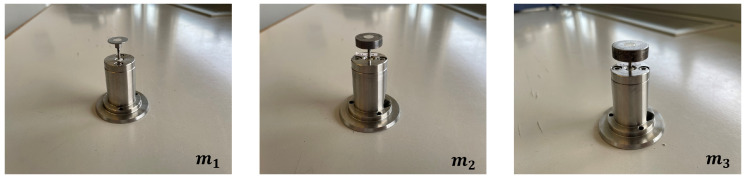
Sensor modules with the newly designed masses and springs.

**Figure 15:**
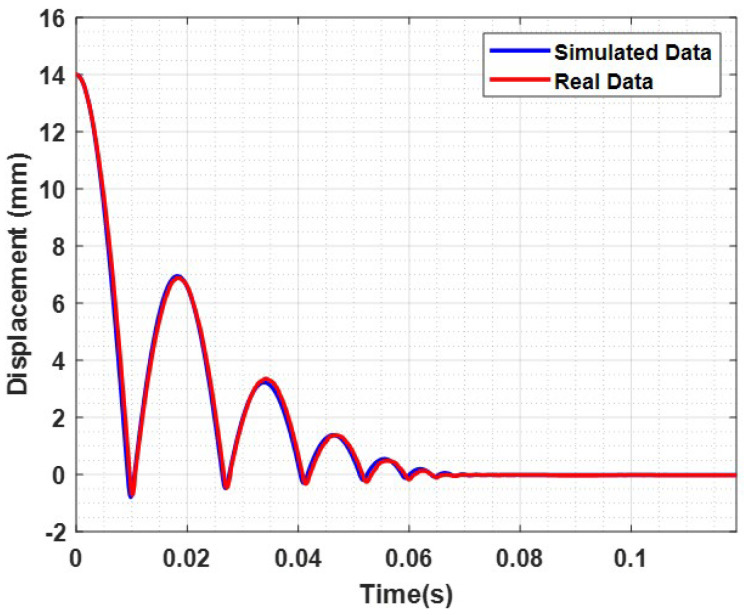
Comparison between simulated data and real acquired data.

**Figure 16:**
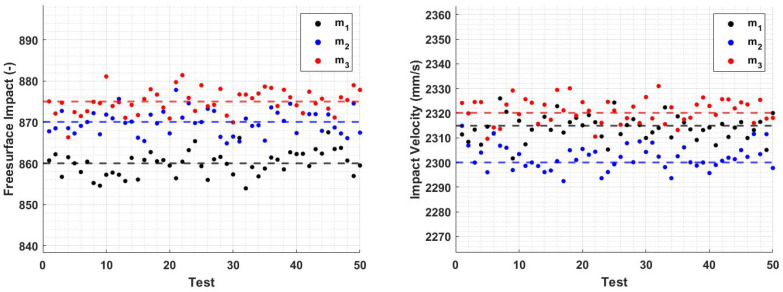
Values of impact-free surface and velocity with the newly designed sensor modules. Mass $m_{1}$ is shown in black, $m_{2}$ is shown in blue, and $m_{3}$ is shown in red.

All obtained results were additionally validated with the real device. To do this, the masses and springs designed following the simulation results were realized and assembled to obtain three different sensor modules (Figure 14). These configurations were used to test a reference material and verify that the results in terms of the impact-free surface and velocity were consistent with the experimental outcomes. Figure 15 shows a comparison between a simulated rebound curve obtained through the virtual Simulink model and a real rebound curve obtained through an experimental test with the VESevo device, choosing in both cases the mass m1. It is evident that the virtual model accurately reproduces the real trend of the rebound curve, giving first proof of its validity. Furthermore, Figure 16 provides evidence that the newly developed sensors guarantee the uniformity of the initial characterization conditions, as predicted in the simulation. In fact, considering a mean value among the 50 tests conducted with the three masses, distinguished by three different colors, both the free surface impact and the impact velocity values change slightly as the mass varies, with a dispersion linked to the causality of the experiment, demonstrating that the redesign of the springs, according to the used mass value, was effective.

## 6. Discussion

The results presented in this study align consistently with the fundamental physical principles governing the VESevo instrument, which relies on the concept of a body experiencing free fall under the influence of gravity and subsequently rebounding off a surface. Let us first examine the influence of mass on rebound curves (Figure 7a). Assuming all other parameters, such as gravity, spring stiffness, spring preload, and internal friction between the rod and guide, remain constant, the results show that the acceleration of the rod is inversely proportional to the mass. This is intuitive, as higher mass increases the system’s inertia, causing it to respond more slowly to applied forces and making it resistant to acceleration or deceleration. This observation is evident in both the falling and ascent phases, resulting in delayed impacts and higher peaks with increased mass. Additionally, heavier rods tend to sink further into the material upon impact due to their greater weight.

The impact of mass on speed follows a similar trend (Figure 7b). Lower accelerations, due to increased mass, lead to reduced impact energy and, thus, lower speed in both the falling and rod–material detachment phases. This is evident from both the lower impact and exit speeds (Figure 8). The variations in storage modulus ($E^{\prime }$) and loss factor (tanδ) shown in Figure 9 are a direct consequence of changes in impact and exit speeds, along with increased sinking depth and time. These factors are all influenced by the increasing mass of the body, impacting the dynamic mechanical behavior of the system and resulting in the observed shifts in material properties. As a result of this analysis, it became evident that compensatory measures were necessary to address the disparities arising from the various masses in the initial conditions. The chosen solution was to modify the elastic element of the system by increasing its stiffness as mass increased. The adjustment in stiffness, made in response to the increased inertia associated with greater mass, effectively compensated for the elevated resistance to motion, which is due to the greater spring reaction. Both the simulated results (Figure 12) and the experimental data (Figure 16) clearly demonstrate the successful application of the stiffness adjustment in preserving uniform impact conditions. Lastly, as the characteristic indentation frequency is inversely related to sinking time, which is observed to increase with mass, it naturally decreases with higher mass. This outcome aligns with the study’s objective of establishing master curves at various frequencies, which will serve as the basis for deriving a shift law.

The physical consistency of the results, as well as the agreement with the experimental results shown in the previous section, validate the virtual model’s accuracy in replicating the VESevo device’s behavior and confirm that the insights drawn from the simulations accurately represent the device’s real-world performance. Therefore, the next step will be to set up a robust experimental test campaign in which, for given polymeric materials, different temperature sweeps will be carried out by employing three different indenter masses. In this regard, one initial limitation of the newly developed system is that the use of three different masses and springs results in extended testing times. This requires replacing the corresponding sensor module each time a test with a different indentation frequency is desired. In the future, more easily interchangeable modules could be designed to assess this challenge. Furthermore, the choice of masses is constrained by overall dimensions. It is crucial to assess whether the selected masses and the consequent indentation frequencies are sufficient, also with experimental data, for establishing clearly separate master curves, from which shift factors can be derived to construct a valid shift law applicable across the entire frequency domain. The expectation is to obtain three distinct master curves, two of which can be appropriately shifted in a specific frequency, chosen as the reference, in order to uniquely identify the temperature dependence of the shift factors for the material under analysis.

## 7. Conclusions

The current configuration of the VESevo device, based on a single indenter mass, lacks the capability to distinctly establish the temperature–frequency relationship of the analyzed viscoelastic material. In order to address this limitation and overcome the problem of a destructive characterization with the DMA, a preliminary simulation study on a validated model was conducted to assess the feasibility of achieving at least three distinct indentation frequencies with the device.

The study confirmed that this objective is achievable, provided that modifications are made to the spring element of the rod–spring system in the VESevo device. These adjustments ensure consistent energy conditions upon impact, ensuring uniform viscoelastic characterization, regardless of the indenter masses employed.

With these promising simulation results, the next step involves conducting a robust experimental testing campaign to characterize viscoelastic materials using three different indenter masses. If the new device setups prove effective in identifying three separate master curves at distinct frequencies, it would create the opportunity to develop a novel shift algorithm based only on non-destructive testing, marking a significant advancement in the characterization of viscoelastic materials. By offering the ability to test polymers in real-time under diverse operating conditions and reconstruct comprehensive master curves, it opens the door to a wide range of applications. The derived viscoelastic properties, in terms of storage modulus and loss factor, can be harnessed for various purposes, such as the development of predictive models for tire grip and wear or to make predictions about the life cycle of a polymer component subject to temperature variations. This methodology not only advances the understanding of polymer behavior but also has the potential to support the development of highly accurate and individually tailored engineering solutions across multiple industries.

## Data Availability

Data are contained within the article.
